# Transcriptome and IgH Repertoire Analyses Show That CD11c^hi^ B Cells Are a Distinct Population With Similarity to B Cells Arising in Autoimmunity and Infection

**DOI:** 10.3389/fimmu.2021.649458

**Published:** 2021-03-19

**Authors:** Robert W. Maul, Michelle D. Catalina, Varsha Kumar, Prathyusha Bachali, Amrie C. Grammer, Shu Wang, William Yang, Sarfaraz Hasni, Rachel Ettinger, Peter E. Lipsky, Patricia J. Gearhart

**Affiliations:** ^1^Laboratory of Molecular Biology and Immunology, National Institute on Aging, National Institutes of Health, Baltimore, MD, United States; ^2^AMPEL BioSolutions LLC, Charlottesville, VA, United States; ^3^RILITE Foundation, Charlottesville, VA, United States; ^4^Research and Early Development, Respiratory and Immunology, BioPharmaceuticals R&D, AstraZeneca, Gaithersburg, MD, United States; ^5^Viela Bio, Gaithersburg, MD, United States; ^6^Lupus Clinical Research Program, Office of the Clinical Director, National Institute of Arthritis and Musculoskeletal and Skin Diseases, NIH, Bethesda, MD, United States

**Keywords:** CD11c, B cells, SLE, autoimmunity, IGH repertoire, B cell transcriptome

## Abstract

A distinct B cell population marked by elevated CD11c expression is found in patients with systemic lupus erythematosus (SLE). Cells with a similar phenotype have been described during chronic infection, but variable gating strategies and nomenclature have led to uncertainty of their relationship to each other. We isolated CD11c^hi^ cells from peripheral blood and characterized them using transcriptome and IgH repertoire analyses. Gene expression data revealed the CD11c^hi^ IgD^+^ and IgD^−^ subsets were highly similar to each other, but distinct from naive, memory, and plasma cell subsets. Although CD11c^hi^ B cells were enriched in some germinal center (GC) transcripts and expressed numerous negative regulators of B cell receptor (BCR) activation, they were distinct from GC B cells. Gene expression patterns from SLE CD11c^hi^ B cells were shared with other human diseases, but not with mouse age-associated B cells. IgH V-gene sequencing analysis showed IgD^+^ and IgD^−^ CD11c^hi^ B cells had somatic hypermutation and were clonally related to each other and to conventional memory and plasma cells. However, the IgH repertoires expressed by the different subsets suggested that defects in negative selection during GC transit could contribute to autoimmunity. The results portray a pervasive B cell population that accumulates during autoimmunity and chronic infection and is refractory to BCR signaling.

## Introduction

A unique subset of B cells that express high levels of CD11c, an integrin, and T-bet (*Tbx21*), a transcription factor, is generated in humans during autoimmunity and chronic infection. In autoimmunity, expanded populations of autoreactive CD11c^hi^ B cells are found in patients with SLE, and they correlate with several measures of disease activity (1). Related B cell subsets in SLE have been described, including CD19^hi^ B cells (2), double negative (DN2) and activated-naïve B cells (3), CD27^−^ memory B cells (4), and CD11c^+^T-bet^+^ B cells (5). Comparable populations associated with other autoimmune diseases include memory CD27^−^IgD^−^ B cells in juvenile idiopathic arthritis (6), CR2/CD21^−^ B cells in rheumatoid arthritis (RA) (7), and CD21^−/low^ B cells in primary Sjogren's syndrome (8), or CD21^lo^ B cells that emerge after immunization with protein antigen (9). Recently, CD11c^+^ CD21^−^ CXCR5^−^ B cells that are expanded in SLE have been shown to correlate significantly to T peripheral helper cells and plasmablast differentiation (10).

In response to chronic infection, a similar B cell subset, termed atypical memory B cells, accumulates in individuals infected with malaria (11), HIV (12), Hepatitis C (13), tuberculosis (14), schistosomiasis (15), and Toxoplasma gondii (16) [reviewed in Karnell et al. (17)]. In addition, these cells may accumulate during acute infection with SARS-CoV-2 infection (18, 19). Although these B cells appear unresponsive to antigen stimulation through the B cell receptor (BCR) (7, 20, 21), it has been argued that they can be stimulated through antigen presented on membranes and differentiate into plasma cells (22).

An analogous population of cells, designated age-associated B cells (ABCs), accumulates in mice during aging, and they also uniquely express CD11c and T-bet (23). Similar to CD11c^hi^ cells in autoimmunity and infection, ABCs are unresponsive to BCR signaling, but can be stimulated through toll-like receptors, such as TLR7 or TLR9 (24). They express a diverse repertoire of heavy and light chain immunoglobulin variable (V) genes that are somatically mutated, suggesting they are a polyclonal, antigen-experienced B cell subset (25). Since ABCs and CD11c^hi^ B cells are associated with autoimmunity in mice (26–28) and humans (1, 3, 29), respectively, it has been suggested that they are functionally equivalent, but their transcriptional relationship has not been examined.

Although these various subsets of B cells share CD11c expression, few efforts to compare them directly have been undertaken. Moreover, their origins and clonal genealogy have not been established. To determine whether these populations are similar, we analyzed transcriptomic signatures to identify gene expression and signaling pathways. Moreover, we evaluated the IgH repertoires of CD11c^hi^ B cells isolated from SLE patients. Together, the data indicate that CD11c^hi^ B cells from SLE patients are a unique population that is closely related to subsets appearing in other autoimmune and infectious diseases.

## Materials and Methods

### B Cell Isolation From SLE Patients

The SLE blood samples were obtained from subjects that met the American College of Rheumatology revised criteria for the classification of SLE and were followed under the Studies of the Pathogenesis and Natural History of Systemic Lupus Erythematosus (SLE) at the National Institute of Arthritis and Musculoskeletal and Skin Diseases of the National Institutes of Health, Bethesda, MD (30). The studies were approved by the Institutional Review Board of the National Institute of Arthritis and Musculoskeletal and Skin Diseases (protocol 94-AR-0066, and 00-AR-0222, respectively). The demographics and clinical characteristics of these SLE blood samples are listed in Supplementary Table 1 (transcriptomic analysis) (1) and Supplementary Table 2 (IgH repertoire analysis). B cell sorting phenotypes are shown in Supplementary Figure 1 have been described previously (1); the purity of the sorted populations was routinely >90%. Peripheral blood B cells from SLE patients (*n* = 4 independent samples) were isolated using a human B cell enrichment kit (StemCell Technologies) and stained as described above. Using a FACS Aria Fusion (BD Biosciences), B cells from SLE patients were sorted as: CD19^+^ CD11c^−^ CD27^−^ IgD^+^ naïve B cells, CD19^+^ CD11c^−^ CD27^+^ IgD^−^ memory B cells, CD19^+^ CD11c^hi^ IgD^+^ B cells or CD19^+^ CD11c^hi^ IgD^−^ B cells.

### Differential Gene Expression

For data sets derived from Affymetrix platforms, GCRMA normalized expression values were variance corrected using local empirical Bayesian shrinkage before calculation of differential expression (DE) using the ebayes function in the open source BioConductor LIMMA package (31). Resulting *p*-values were adjusted for multiple hypothesis testing and filtered to retain DE probes with a False Discovery Rate (FDR) < 0.05 (32). For RNAseq datasets, FASTQC files were obtained from GEO. Trimmomatic was performed to cut adapter sequences, low quality reads, and the first six reads for each sequence because of non-random primer bias. Reads were aligned to human reference genome hg38 in STAR with default parameters, and then SAM files were converted to BAM files using Sambamba. Relative differential expression counts were generated using featureCounts. After careful examination with PCA, one outlier RA patient from GSE110999 was removed. Data were normalized using DeSeq2 bioconductor R package. Differentially expressed gene (DEG) comparisons between various cell populations were done using generalized linear modeling from the DeSeq2 package. Resulting *p*-values were adjusted for multiple hypothesis testing and filtered to retain DEG probes with an FDR < 0.05. FastQC, Trimmomatic, STAR, Sambamba, and the featureCounts programs are all free, open source programs available at the following web addresses:

FastQC, https://www.bioinformatics.babraham.ac.uk/projects/fastqc/;
Trimmomatic, http://www.usadellab.org/cms/?page=trimmomatic;
STAR, http://labshare.cshl.edu/shares/gingeraslab/www-data/dobin/STAR/Old/Releases/STAR_2.4.0h1/doc/STARmanual.pdf;
Sambamba, http://lomereiter.github.io/sambamba/; and
FeatureCounts, http://subread.sourceforge.net/

Exploratory analysis like Principal Component Analysis (PCA) was conducted on the DeSeq2 normalized counts using R Bioconductor package factoextra (v1.0.7). The arguments addEllipses, ellipse.type, ellipse.level, were used to draw stat ellipses around each cell type at 95% confidence level in order to determine any outlier samples. The top 1,000 most differentially expressed genes were used for PCA analysis.

### Gene Clustering

The database for annotation, visualization and integrated discovery (DAVID) (http://david.abcc.ncifcrf.gov/) was used to determine enriched gene ontology (GO) biological pathways (BP) for increased or decreased human or mouse gene symbols. BIG-C, a functional clustering tool that sorts increased and decreased genes into 52 categories based on their most likely biological function and/or cellular localization, was also employed. The BIG-C is based on information from multiple online tools and databases including UniProtKB/Swiss-Prot, GO Terms, MGI database, KEGG pathways, NCBI PubMed, and the Interactome. Each gene is placed into only one category based on its most likely function to eliminate the redundancy in enrichment, sometimes found in GO BP annotation (33).

### Gene Set Variation Analysis (GSVA)

GSVA (V1.25.0) was used as a non-parametric, unsupervised method for estimating the variation of pre-defined gene sets in samples of microarray expression data sets (www.bioconductor.org/packages/release/bioc/html/GSVA.html) (34). The inputs for the GSVA algorithm were a gene expression matrix of log2 expression values for pre-defined gene sets co-expressed in SLE datasets (Supplementary Data File 1). Enrichment scores (GSVA scores) were calculated non-parametrically using a Kolmogorov Smirnoff (KS)-like random walk statistic and a negative value for a particular sample and gene set, meaning that the gene set has a lower expression than the same gene set with a positive value. The enrichment scores (ES) were the largest positive and negative random walk deviations from zero, respectively, for a particular sample and gene set. The positive and negative ES for a particular gene set depend on the expression levels of the genes that form the pre-defined gene set. GSVA calculates enrichment scores using the log2 expression values for a group of genes in each SLE patient and healthy control and normalizes these scores between −1 (no enrichment) and +1 (enriched).

### Hierarchical Clustering

Morpheus (Morpheus, https://software.broadinstitute.org/morpheus) was used for unsupervised hierarchical clustering analysis. The inputs for Morpheus were the DEGs (log fold change) for each B cell population compared to either naïve or memory B cells. The distance measure was one minus Pearson's correlation coefficient with average linkage and clustering set to both rows and columns.

### Comparison of Mouse to Human Gene Expression Data

The Bioconductor package entitled homologene (v1.1.68, https://www.rdocumentation.org/packages/homologene/versions/1.1.68) was used for conversion of mouse gene symbols to their human ortholog gene symbols. Mouse gene symbols which did not convert to a human ortholog equivalent were entered manually into the Mouse Genomic Informatics database (informatics.jax.org) to check if there was a human homolog.

### IgH Repertoire Analysis

RNA from eight SLE patients (Supplementary Table 2) was isolated by resuspending cell pellets in 100 μL PBS and adding 1 mL TRIzol reagent (Thermofisher Scientific). 200 μL chloroform was added to each sample, mixed, and centrifuged at 12,000 rfc to separate RNA (aqueous phase) from DNA (interphase). RNA was precipitated with 2 μL polyacyl carrier and 500 μL isopropanol, followed by centrifugation and resuspension in 25 μL RNAase-free water. cDNA was generated using Superscript III enzyme (Thermofisher Scientific) per manufacturer's recommendation. IgH transcripts were amplified with Herculase II Fusion DNA Polymerase (Agilent) and leader (L) primers for each V_H_ family and primers for each C_H_ gene: 5′L-VH1; ACA GGT GCC CAC TCC CAG GTG CAG, 5′L-VH3; AAG GTG TCC AGT GTG ARG TGC AG, 5′L-VH4/6; CCC AGA TGG GTC CTG TCC CAG GTG CAG, and 5′L-VH5; CAA GGA GTC TGT TCC GAG GTG CAG (35) that were paired with constant gene primers: hIgM; GGC CAC GCT GCT CGT ATC C, hIgD; GAC CAC AGG GCT GTT ATC CTT TGG, hIgG; CGC CTG AGT TCC ACG ACA CC, and hIgA; GGA AGA AGC CCT GGA CCA GGC. PCR products were cloned into pSC-A-amp/kan cloning vector (Agilent) and Sanger sequenced. IgH VDJ sequence identity and mutational abundance were identified using IMGT database (http://www.imgt.org/). Clonal sequences were identified as having the same V- and J-gene segments with identical CDR3 nucleotide sequences. The use of family specific V-gene primers was chosen to allow for Sanger sequencing to minimize errors generated during other forms of IgH amplification. This technique will introduce minor amplification bias into the analysis; however, the amplification bias will be identical for all populations analyzed.

### Statistics

GraphPad PRISM 8 version 8.2.1 was used to perform mean, median, mode, standard deviation, and Tukey's multiple comparisons test, The Fisher's exact test was performed in R. For IgH repertoire analysis, two-tailed *T*-test was performed to examine V-gene usage difference.

### Gene Expression Datasets and Data Sharing Statement

RNA-seq and microarray datasets, with accession numbers GSE110999, GSE13917, GSE92387, GSE65928, GSE69033, GSE68878, and GSE28887, are available on the NCBI's database Gene Expression Omnibus (GEO) (https://www.ncbi.nlm.nih.gov/geo/). The FCRL4^+^ tonsil B cell microarray data (formerly listed as CBX-40 at CIBEX (36) is available at ftp://ftp.ddbj.nig.ac.jp/ddbj_database/gea/experiment/E-GEAD-000/E-GEAD-336/. Supplementary Data File 2 lists gene expression dataset details including sorted populations and platforms.

## Results

### CD11c^hi^ B Cells Are a Distinct Population Compared to Naïve or Memory B Cells

**Figure 1:**
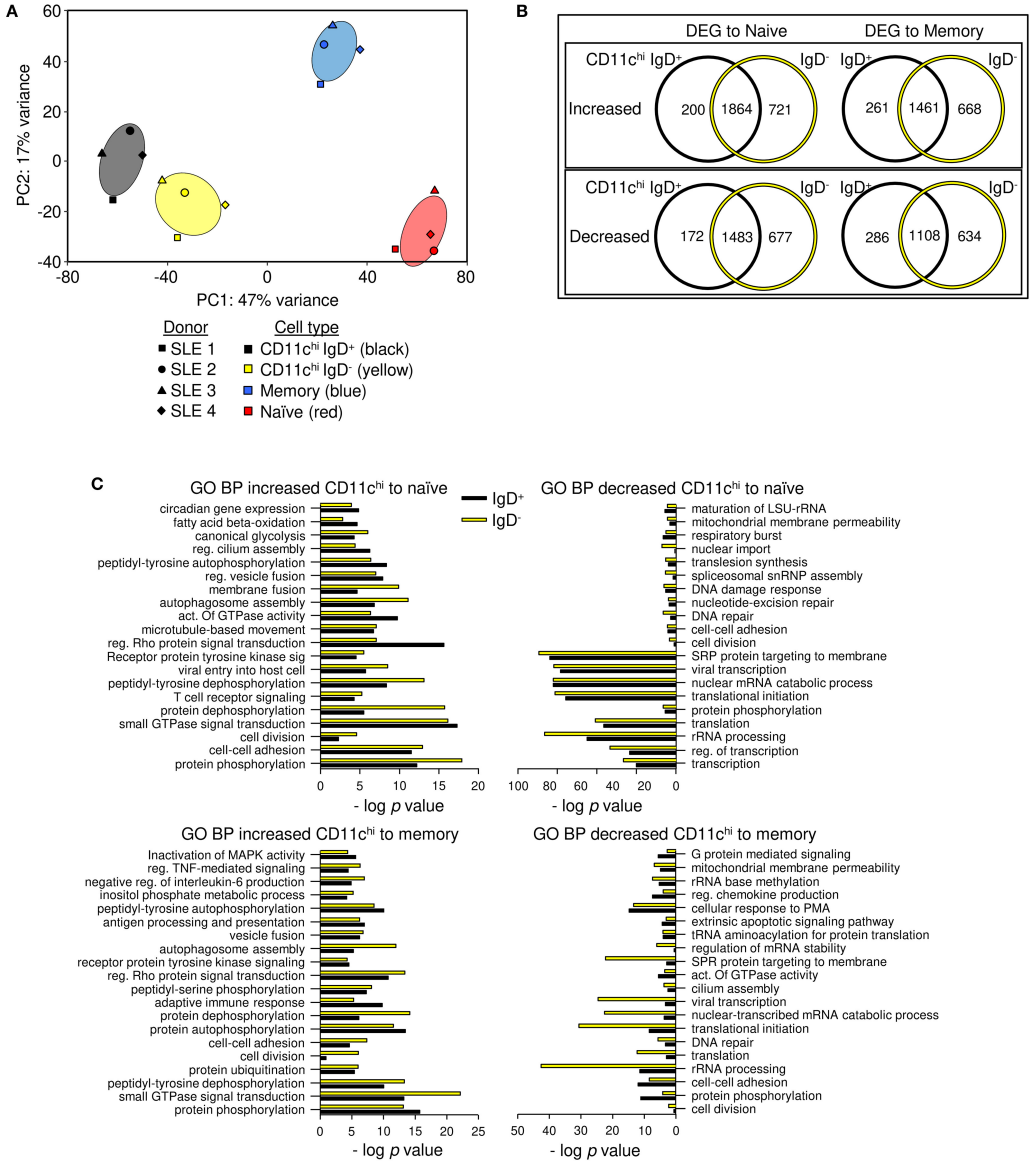
CD11c^hi^-IgD^+^ and IgD^−^ B cells have similar gene expression and are different from memory and naïve B cells. **(A)** Principal component analysis of CD19^+^ CD11c^hi^ IgD^+^ CD27^−^ (black), CD19^+^ CD11c^hi^ IgD^−^ CD27^−^ (yellow), CD19^+^ IgD^−^ CD27^+^ (memory, blue), and CD19^+^IgD^+^CD27^−^ (naïve, red) B cells. Ellipses demonstrate the 95% confidence intervals. **(B)** Limma DEG analysis was done separately for CD11c^hi^ IgD^+^ and CD11c^hi^ IgD^−^ compared to either naïve or memory B cells (FDR < 0.05). Genes are listed in Supplementary Data File 3. **(C)** GO BP analysis was used to determine functional enrichment of increased or decreased transcripts. Pathways with the lowest *p*-values are graphed by -log *p* value. Complete list of GO BP and genes in each pathway are in Supplementary Data File 6.

Previous reports suggested there might be two subpopulations of CD11c^+^ cells in SLE patients based on IgD expression: IgD^+^ activated naïve cells that produce autoantibodies, and IgD^−^ double negative cells (CD27^−^ IgD^−^, DN2) that are poised to become plasma cells (3, 37, 38). To examine how different they are, we sorted CD11c^hi^ IgD^+^ and IgD^−^ cells and compared their transcriptional relatedness to naïve and conventional memory B cells. Using deposited RNAseq data (Supplementary Data File 2) from SLE patients (Supplementary Table 1) (1), principal component (PC) analysis revealed that CD11c^hi^ IgD^+^ and IgD^−^ may be separate populations, but they are vastly different from naive (CD11c^−^ CD27^−^ IgD^+^) and memory (CD11c^−^ CD27^+^ IgD^−^) B cells (Figure 1A). PC1 represented 47% of the variance between populations and separated naïve and memory cells from the IgD^+^ and IgD^−^ B cells, and PC2 represented 17% of the variance between populations and separated memory from naïve B cells. When DEGs from CD11c^hi^ IgD^+^ or IgD^−^ B cells were compared to naïve or memory B cells, the majority of genes were shared between the two subsets (Figure 1B, Supplementary Data File 3). Many DEGs encoding immune-related cell surface and signaling proteins were detected in both CD11c^hi^ IgD^+^ and IgD^−^ B cells in comparison to naive and/or memory B cells (Table 1), emphasizing both the uniqueness of the total CD11c^hi^ population and the similarity of the IgD^+^ and IgD^−^ subsets. Although only four samples were analyzed, the overall similarity of the results suggested that the populations were highly representative.

**Table 1:** Immune related transcripts that define CD11c^hi^ B cells.

**Increased in CD11c**^****hi****^ **to naïve and memory**			**Increased in CD11c^hi^ to naïve**		**Increased in CD11c^hi^ to memory**
*ADGRE2*	*ADGRE5*	*ADGRG5*	*ADGRE3*		*BACH1*
*AICDA*	*BATF*	*BCL11B*	*ANPEP*		*CCDC88B*
*BCL6*	*CARD11*	*CD164*	*CD226*		*CD22*
*CD19*	*CD200R1*	*CD274*	*CD80*		*CD180*
*CD72*	*CD86*	*CLEC1*	*CXCR3*		*CD200*
*CREM*	*CSK*	*CTSW*	*FAS*		*IL21R*
*DAPP1*	*DOK1*	*DTX1*	*LILRB4*		*LAIR1*
*EFHD2*	*FCGR2A*	*FCGR2B*	*LPXN*		*PAX5*
*FGR*	*GZMM*	*GRAP*	*LSP1*		*PIK3AP1*
*HCK*	*HCST*	*IL10RA*	*LTBR4*		*RASGRP3*
*IL10RB*	*IL12RB2*	*KIR3DX1*	*PAG1*		*RUNX1*
*LCP2*	*LILRA1*	*LILRB1*	*PRDM1*		*ST6GAL1*
*LILRB2*	*LRMP*	*LY96*	*SIGLEC5*		
*NKG7*	*P2RX7*	*PDCD1*	*TNFRSF13B*		
*PDCD1LG2*	*POU2F2*	*RELT*	*VAV1*		
*RFTN1*	*RGS13*	*S1PR5*			
*SEMA7A*	*SIGLEC6*	*SIGLEC10*			
*SIK1*	*SKAP2*	*SLAMF1*			
*SLAMF7*	*SRGN*	*STAT3*			
*STK10*	*SYK*	*TBX21*			
*TFEB*	*THEMIS2*	*TNFRSF1B*			
*TYROBP*	*UBASH3B*	*XBP1*			
**Decreased in CD11c^hi^ to naïve and memory**			**Decreased in CD11c^hi^ to naïve**		**Decreased in CD11c^hi^ to memory**
*CCR7*	*CD6*	*CD24*	*BACH2*	*BTLA*	*CAMK4*
*CD40*	*CD44*	*CLEC17A*	*CD1A*	*CD200*	*CCR2*
*CR1*	*CR2*	*CXCL16*	*CD38*	*CD69*	*CD27*
*CXCR4*	*CXCR5*	*DCK*	*CD79B*	*ETS1*	*CD70*
*FAM129C*	*FCER2*	*GNG7*	*GIMAP2*	*ICAM2*	*GIMAP8*
*HIF1A*	*HIVEP2*	*HLX*	*IL4R*	*STAP1*	*ICAM1*
*IL13RA1*	*JAK3*	*KLHL6*	*SWAP70*	*TCL1A*	*IL6R*
*LAMP3*	*LCK*	*MZB1*			*LAX1*
*MYC*	*NFAM1*	*P2RY8*			*TCF7*
*PIM2*	*POU2F1*	*PRDM4*			
*SMAD3*	*SPI1*	*TNFRSF18*			
*TNFSF8*	*VPREB3*				

GO BP analysis of the differentially expressed transcripts compared to naïve or memory B cells, demonstrated that CD11c^hi^ IgD^+^ and IgD^−^ B cells had similarly enriched functional categories, in agreement with there being few differentially expressed transcripts between the two populations isolated based on IgD expression (Figure 1C, Supplementary Data File 4). A second functional clustering program, biologically informed gene clustering (BIG-C) (33), also demonstrated agreement with enriched categories for CD11c^hi^ IgD^+^ and IgD^−^ B cells compared to naïve and memory B cells, with notable differences of enrichment in autophagy in IgD^+^ B cells and endosome and vesicles in IgD^−^ B cells when compared to naïve B cells, but no significant differences when compared to memory B cells (Supplementary Figure 2A, Supplementary Data File 5). The functional overlap was further corroborated by the similarity in the expression of hundreds of pathways by CD11c^hi^ IgD^+^ and IgD^−^ B cells using Ingenuity Pathway Analysis (IPA) (Supplementary Figure 2B). Out of 410 pathways, a few cell cycle related pathways had a Z-score difference of more than 1 between IgD^+^ and IgD^−^ cells, with IgD^−^ having lower, negative scores, although not reaching Z score values of −1.8 for true significance. Additionally, mTor and complement signaling related pathways had a Z score difference of more than 1 between IgD^+^ and IgD^−^ cells; these scores were higher in IgD^−^ B cells and were significant.

Dendrogram grouping of transcripts showed intermingling of CD11c^hi^ IgD^+^ and IgD^−^ B cells and a similar distance of these populations from memory and naive B cells, suggesting patient-specific differences were more important than IgD expression by CD11c^hi^ cells, and supporting the overlap in DEGs when compared to naïve and memory B cells (Supplementary Figure 2C). Whereas, comparison of CD11c^hi^ IgD^+^ or IgD^−^ B cells to either naïve or memory B cells detected thousands of DEGs, there was a difference of only 175 DEGs when CD11c^hi^ IgD^+^ and IgD^−^ cells were compared to each other (Supplementary Data File 6). Pathway analysis using GO BP determined that the CD11c^hi^ IgD^+^ B cells had increased expression of genes associated with the respiratory burst and cell adhesion, whereas IgD^−^ B cells expressed increased transcripts for intracellular signal transduction, microtubule organization, and positive regulation of transcription (Supplementary Data File 7). Taken together, the CD11c^hi^ IgD^+^ and IgD^−^ B cells are highly similar and potentially consist of a single population with variable levels of IgH class switch recombination.

### CD11c^hi^ B Cells Are Enriched in Transcripts Related to Inhibition of B Cell Signaling

**Figure 2:**
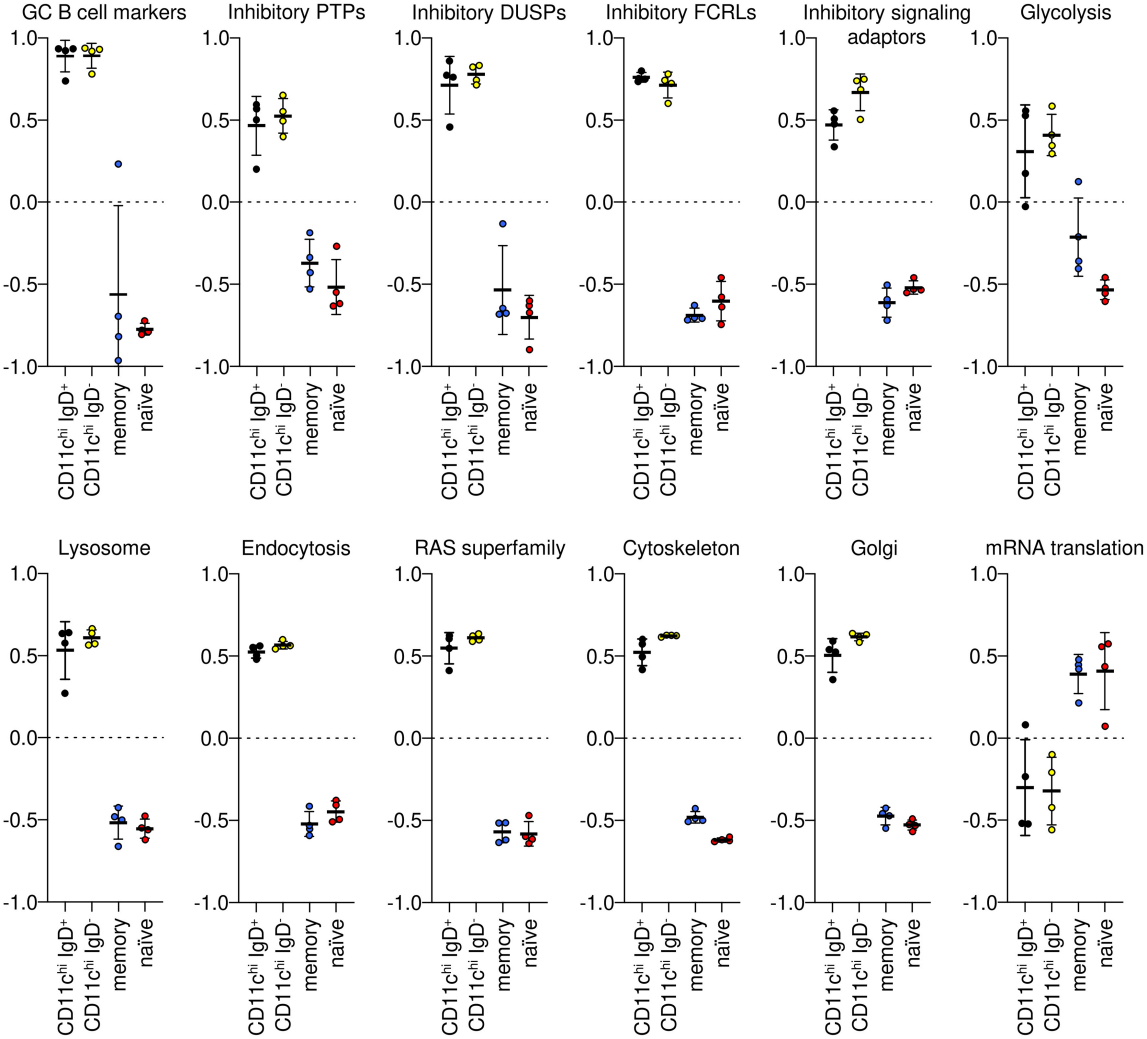
GSVA of gene signatures enriched in CD11c^hi^ B cells by GO BP and BIG-C analysis. IgD^+^ and IgD^−^ B cells were not significantly different from each other, but they were significantly different (Tukey's multiple comparisons test *p* < 0.0001) from memory and naïve B cells for each signature. Gene symbols for each GSVA enrichment module are in Supplementary Data File 1.

Gene set variation analysis (GSVA) was used to examine the co-expression of groups of genes identified as increased or decreased in CD11c^hi^ B cells compared to both naïve and memory B cells (Supplementary Data Files 3–5). A few germinal center (GC) B cell markers were uniformly increased in both CD11c^hi^ IgD^+^ and IgD^−^ B cells compared to naïve and memory B cells. Inhibitory protein-tyrosine phosphatases (PTPs), inhibitory dual-specific phosphatases (DUSPs), inhibitory Fc receptor-like (FCRLs), and inhibitory signaling adaptors were also significantly enriched in CD11c^hi^ IgD^+^ and IgD^−^ cells compared to naïve and memory B cells, as were genes associated with glycolysis. Likewise, gene signatures for lysosome, endocytosis, Ras superfamily of GTPases, cytoskeleton, and Golgi were uniformly enriched in CD11c^hi^ B cells compared to naïve and memory B cells. However, genes involved in mRNA translation were significantly decreased in both CD11c^hi^ IgD^+^ and IgD^−^ (Figure 2). Uniform enrichment of groups of inhibitory signaling molecules in CD11c^hi^ IgD^+^ and IgD^−^ B cells has not previously been described and may suggest an interrupted or inhibited signaling process.

### CD11c^hi^ B Cells Are a Distinct B Cell Population

Our transcriptional analysis (Table 1) showed that CD11c^hi^ IgD^+^ and IgD^−^ B cells from SLE patients have decreased expression of *CR2* (CD21), *CD27*, and *CXCR5* consistent with B cell populations from RA and combined variable immunodeficiency (CVID) described in the literature as anergic (7) (CD21^−^ CD27^−^), DN2 from SLE patients (3) (CD27^−^ IgD^−^ CXCR5^−^), atypical from malaria patients (21) (CD21^−^ CD27^−^), and FCRL4^+^ tissue memory B cells from tonsil (36). Additionally, GC B cell markers *AICDA, BCL6, DAPP1*, and *RGS13* were increased in both CD11c^hi^ IgD^+^ and IgD^−^ cells compared to naïve and memory (Table 1), which suggested this population could be closely related to GC B cells. However, genes for plasma cell markers *XBP1* and *SLAMF7* were also increased in both IgD^+^ and IgD^−^ subsets compared to naïve and memory B cells, implying CD11c^hi^ B cells might display an intermediate phenotype with both GC and plasma cell markers.

**Figure 3:**
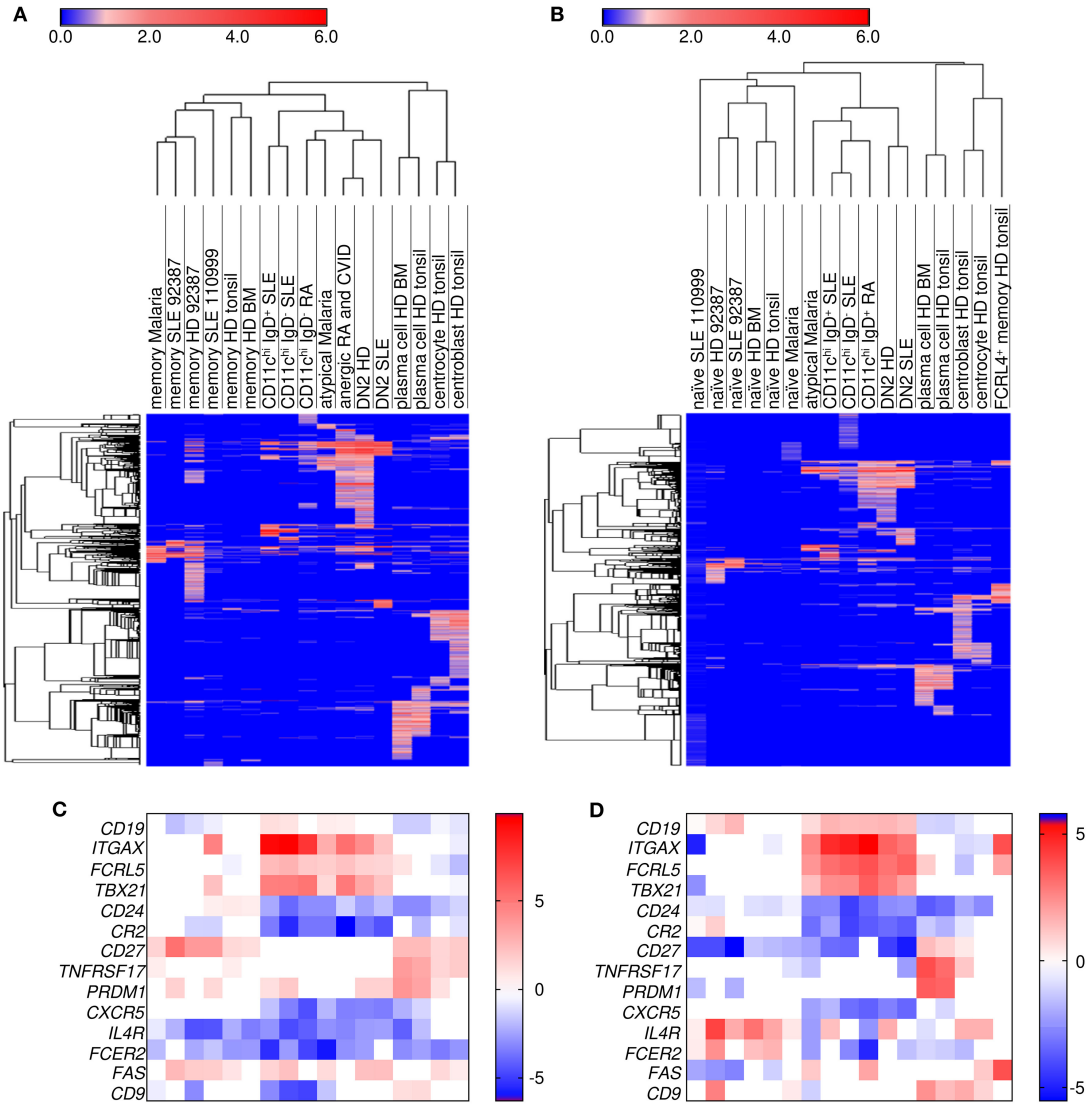
Hierarchical clustering demonstrated that CD11c^hi^ B cells are most similar to DN2, CD21^−^ CD27^−^ (atypical), and CD10^−^ CD21^−^ CD27^−^ (anergic) B cells. Limma differential expression analysis was performed between cell populations from multiple datasets to either naïve **(A)** or memory **(B)** B cells included in each dataset (Supplementary Data File 8). Transcripts increased in each cell population compared to naïve or memory cells at an FDR < 0.05 were used for row and column hierarchical cluster analysis (one minus Pearson correlation) in Morpheus (https://software.broadinstitute.org/morpheus). The color numerical scale represents the log fold change to naïve **(A)** or memory **(B)**. HD, healthy donor; BM, bone marrow. Analysis of a select group of genes which separate the cell populations are shown for naïve **(C)** or memory **(D)** B cells. *ITGAX*, CD11c; *TBX21*, T-bet. The order of the cell populations in the heatmaps is the same as the hierarchical clustering maps above each heatmap.

To determine whether these B cells are a common cell population which arise under different stimuli, we compared gene expression profiles of the above populations with naïve, memory, centroblasts, centrocytes, plasmablasts from tonsil, and plasma cells from bone marrow (39). As can be seen in Figure 3, CD11c^hi^ B cell transcripts from SLE and RA patients clustered most closely with DN2 (SLE), DN2 (HD), anergic (RA/CVID), and atypical (malaria) B cells compared to either naive (Figure 3A) or memory (Figure 3B) B cells from each data set. Hierarchical clustering also demonstrated that the CD11c^hi^ cell subset shared more transcripts in common with either naïve or memory B cells, and fewer transcripts in common with GC centroblasts, centrocytes, and plasma cells, even though the CD11c^hi^ B cells expressed some common GC or plasma cell transcripts when compared to naïve or memory B cells. Of note, FCRL4^+^ tissue memory cells (tonsil) grouped most closely with centrocytes and centroblasts despite over-expressing *ITGAX* (CD11c). Compared to naïve or memory B cells, the DEGs in centroblasts and centrocytes were associated with DNA repair, chromatin remodeling, and cell cycle genes, but very few of these transcripts were increased in the CD11c^hi^ B cell subsets when compared to naïve and memory B cells. Similarly, compared to naïve and memory B cells, the DEGs in plasma cells were linked to Ig secretion, unfolded protein response, proteasome, endoplasmic reticulum and Golgi genes, but these gene were not expressed in the CD11c^hi^ B cell subsets compared to memory and naïve B cells (Supplementary Data File 8). Hierarchical clustering demonstrated that cell populations referred to as atypical (malaria), anergic (RA/CVID) and DN2 (SLE) were most closely related to the CD11c^hi^ B cell populations and were separated from all other B cell populations. Notably, the CD11c^hi^-like populations all shared increased expression of *ITGAX* (CD11c), *FCLR5*, and *TBX21* (T-bet) along with down regulation of *CR2* (CD21) and *CD27* compared to naïve (Figure 3C) and memory (Figure 3D). We have previously observed that CD11c^hi^ cells arise in healthy individuals, albeit at a lower frequency than during SLE (1). Analysis of the DN2 population from healthy donors showed close clustering with the CD11c^hi^-like populations from various diseases. This suggested that the population can arise under a variety of circumstances and is not limited to SLE.

**Figure 4:**
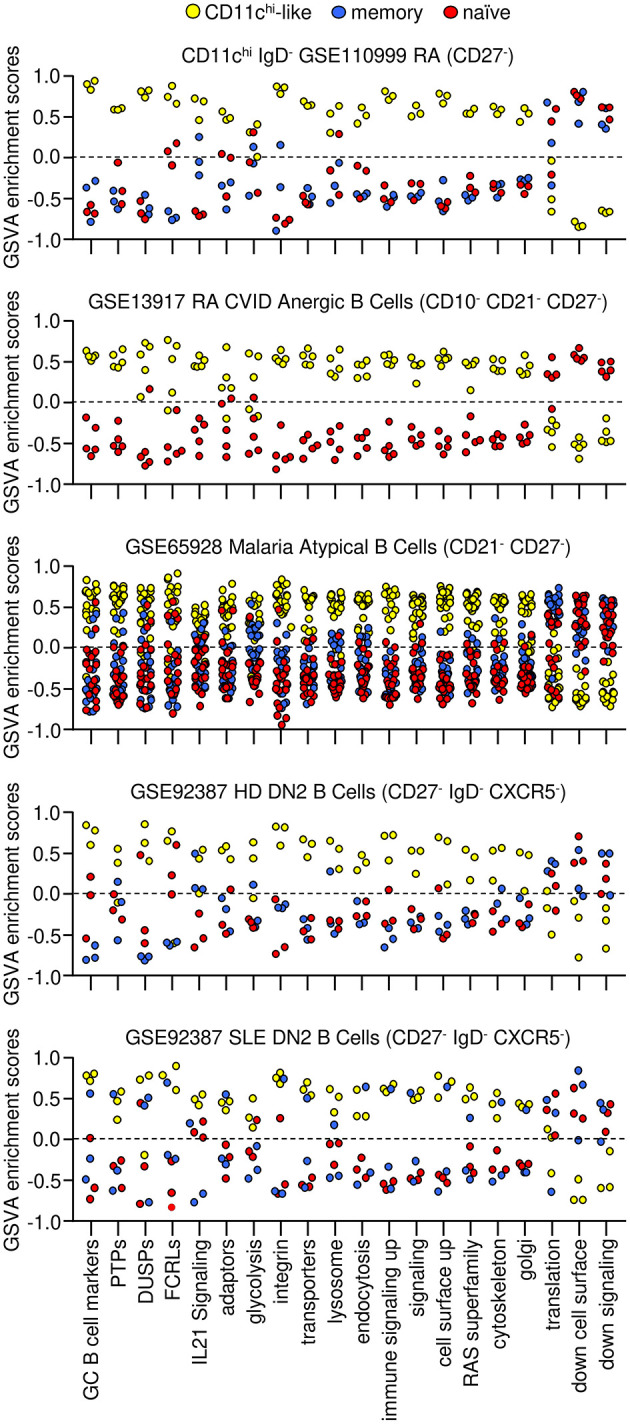
CD11c^hi^-like, memory, and naive gene expression signatures from B cells isolated with different markers from patients with different diseases. Enriched signatures determined in CD11c^hi^ B cells from SLE patients (yellow circles) were used for GSVA of B cell populations isolated by low CD21 and/or low CD27 expression to determine similarities to CD11c^hi^ B cells. Decreased signatures are shown for memory (blue) and naïve (red) cells. GSVA was carried out on datasets from RA patients (*n* = 3 of each: CD11c^hi^ IgD^−^, naïve, memory), RA and CVID patients (*n* = 5 of each: anergic, naïve), malaria patients (*n* = 20 atypical, *n* = 19 naïve, *n* = 20 memory), HD (*n* = 3 of each: DN2, naïve, memory), and SLE patients (*n* = 3 of each: DN2, naïve, memory). Tukey's multiple comparisons test was performed between B cell populations for each GSVA enrichment category of each dataset, and *p*-values to naïve and memory B cells are listed in Supplementary Data File 9.

To understand how the CD11c^hi^ IgD^−^-like populations are related, GSVA was used to examine enrichment of gene sets that were detected in CD11c^hi^ B cells from SLE. Strikingly, B cell populations isolated on the basis of low IgD, CD27, CD21, and CXCR5 had similar GSVA enrichment patterns as CD11c^hi^ B cells. All five populations had increased GC marker expression and high expression of inhibitory PTPs, DUSPs, FCRLs and signaling adaptors compared to naïve and memory B cells. These individual B cell subsets expressed increased integrin, transporter, lysosome, endocytosis, RAS superfamily, cytoskeleton, and Golgi signatures similar to CD11c^hi^ B cells from SLE patients. Furthermore, genes representative of IL-21 signaling, previously demonstrated to be increased in CD11c^hi^ B cells from SLE patients (1), were increased in these B cell subsets as well. However, transcripts for mRNA translation and cell surface/signaling transcripts decreased in CD11c^hi^ IgD^+^ and IgD^−^ cells from SLE patients were not universally enriched in all the CD11c^hi^-like B cells, compared to naïve and memory B cells suggesting that these differences may be specifically related to SLE (Figure 4, Supplementary Data File 9). Notably, all subsets had increased *ITGAX, TBX21, SLAMF7, FCRL5* and *SYK* and decreased *CCR7, CD24, CR2, CXCR4, CXCR5*, and *IL4R* compared to naïve and memory B cells.

### Mouse ABCs Are Transcriptionally Distinct From Human CD11c^hi^ Cells

**Figure 5:**
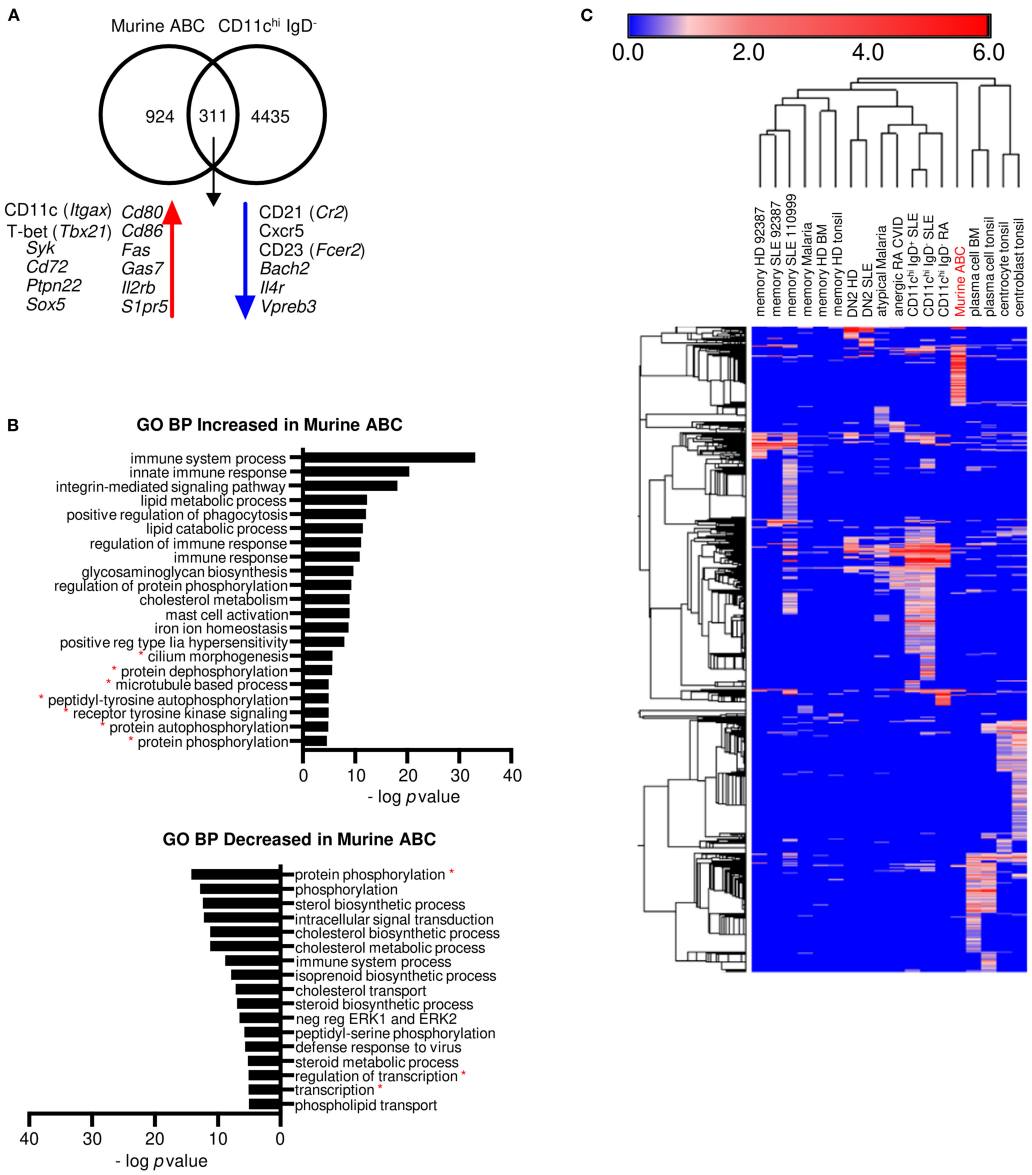
Marker genes and a few pathways are similar between human CD11c^hi^ IgD^−^ B cells and mouse ABCs, but ABCs are mostly distinct from the human cells. **(A)** Transcripts in common and either increased or decreased between murine ABC DE to murine FO B cells (GSE28887) and human CD11c^hi^ IgD^−^ B cells DE to human naïve B cells (GSE110999). Murine gene symbols were translated to human orthologs and compared to human gene symbols (gene symbols in Supplementary Data File 10). **(B)** GO BP analysis of increased and decreased transcripts of murine ABC compared to murine FO B cells. Red asterisks indicate pathways in common with human CD11c^hi^ B cells. Mouse gene symbols for increased or decreased genes were entered directly into DAVID for GO BP analysis and enriched categories with genes listed are in Supplementary Data File 11. **(C)** Hierarchical clustering of the log fold change for increased genes from each human cell type DE to naïve human B cells, and murine ABC cells DE to murine FO B cells. Murine genes were converted to human orthologs for this analysis. The color numerical scale represents the log fold change to naïve B cells. Morpheus (https://software.broadinstitute.org/) was used for hierarchical clustering. Gene symbols and LFC are listed in Supplementary Data File 12.

A population in mice called age-associated B cells (ABCs) is considered by some to be the equivalent of the human DN2 cell population, and the human and murine populations are considered singular in some review articles (40, 41). ABCs are B220^+^CD19^+^CD11b^+^ spleen cells that also may express CD11c, but they are not isolated in a similar manner to any of the human populations. To determine the relationship of mouse splenic ABCs to human peripheral blood CD11c^hi^ B cells, DE analysis of the T-bet^+^ (*Tbx21*) CD11b^+^ (*Itgam*) CD11c^+^ (*Itgax*) murine ABC subset compared to the mouse follicular (FO) B cell subset from murine spleen of C57BL/6 mice (GSE28887) (27) was carried out. Mouse genes were then converted to their human orthologs, and the DEGs were compared to those of human CD11c^hi^ B cells differentially expressed to naïve B cells because healthy mice have mostly naïve B cells. The ABC population shared 311 transcripts with human CD11c^hi^ IgD^−^ B cells, including increased *Itgax, Tbx21, Syk, Cd72, Ptpn22*, and *Sox5*, and decreased *Cr2, Cxcr5*, and *Fcer2* (Figure 5A, Supplementary Data File 10), but had no increased expression of GC markers. Seven GO BP categories were in common with human CD11c^hi^ B cells: cilium morphogenesis, protein dephosphorylation, microtubule-based process, peptidyl-tyrosine autophosphorylation, receptor protein tyrosine kinase signaling, protein autophosphorylation, and protein phosphorylation. However, GO BP categories with the most significant enrichment in mouse ABCs, such as immune system process, innate immune signaling, integrin signaling, and regulation of phagocytosis, were not enriched in human CD11c^hi^ B cells (Figure 5B, Supplementary Data File 11). Hierarchical clustering analysis using human orthologs of murine genes that were increased in ABCs compared to murine FO B cells showed that mouse ABCs were equally distant from all human B cell subsets (Figure 5C, Supplementary Data File 12). A deeper analysis for potential inhibitors of B cell signaling in ABCs revealed an increase in *Siglece, Ptpn22, Cd72, PirB, Pilra, Cd5* and *Lair1*, suggesting some similarity to the large number of inhibitory signaling genes increased in CD11c^hi^-like B cells, but the mouse ABC subset also expressed an increase in multiple cytokines and chemokines, including *Il18, Cxcl9, Ccl9, Ccl8, Ccl6, Cxcl10, Il6, Tnfsf13b*, and *Cxcl13* not detected as increased in any of the human CD11c^hi^-like populations. The increased integrin pathways in mouse ABC could reflect their origin as tissue resident cells and may not be an appropriate comparator to human circulating cells. Overall, these analyses suggest that mouse ABCs are distinct from human CD11c^hi^-like populations, and caution should be used in interpreting them as equivalent from a functional standpoint at this time.

### IgH V Gene Repertoires of CD11c^hi^ B Cells and Naïve B Cells Are Similar

**Figure 6:**
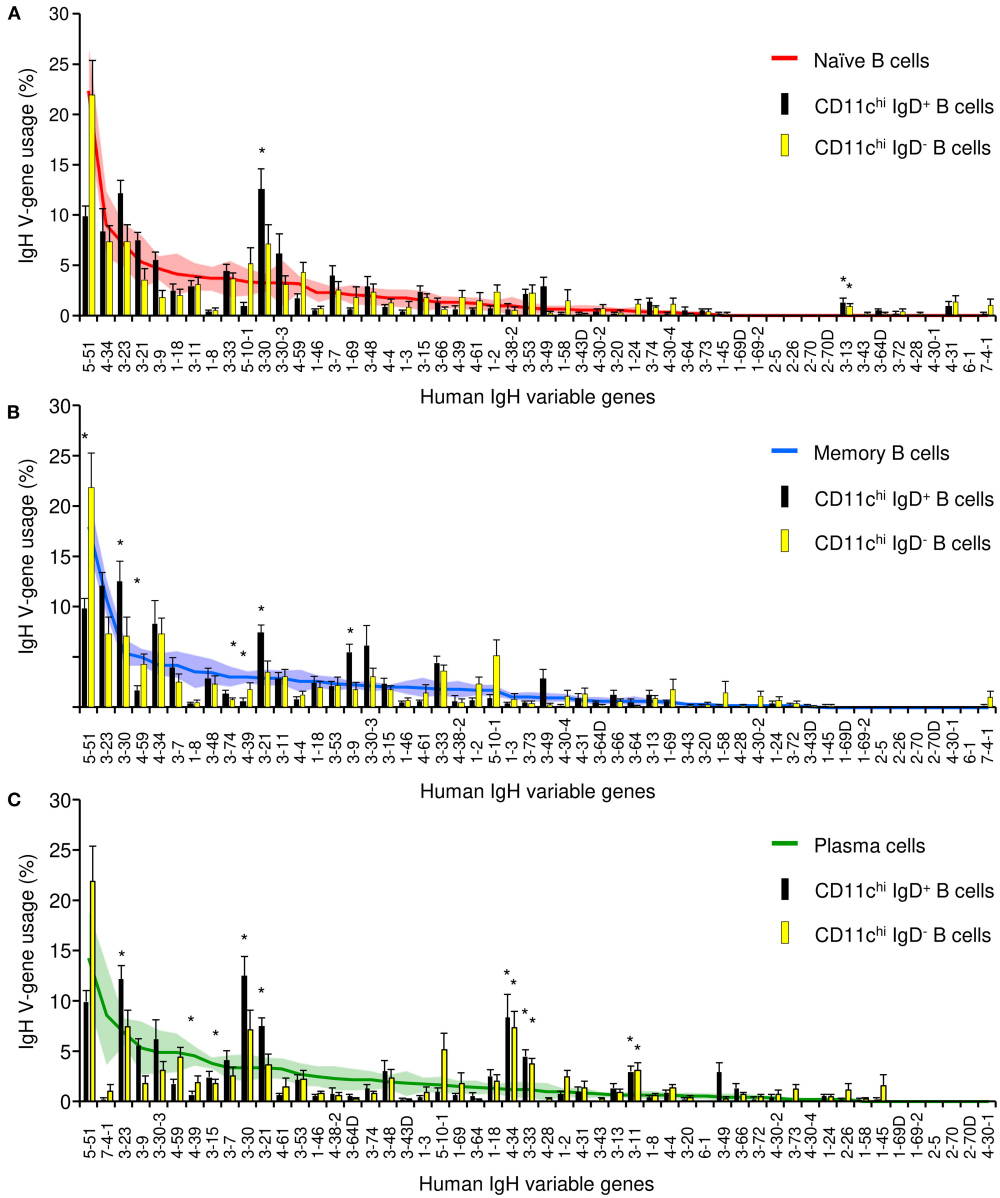
IgH repertoire analysis of CD11c^hi^ IgD^+^ and IgD^−^ B cells compared to average V-gene usage in naïve, memory, and plasma cells. Bars represent the average V-gene usage in CD11c^hi^ IgD^+^ (black) and CD11c^hi^ IgD^−^ (yellow) B cells. Lines represent the average V-gene usage in naïve (**A**, red), memory (**B**, blue), or plasma cells (**C**, green). Shaded area and error bars represent the standard error of the mean; *represents *p* < 0.05 by Fisher exact test.

We next compared the repertoires of expressed V_H_ genes of five populations of B cells that were sorted from eight SLE patients: CD11c^hi^ IgD^+^, CD11c^hi^ IgD^−^, CD11c^−^ CD27^−^ naïve, CD11c^−^ CD27^+^ IgD^−^ memory, and CD27^+^ CD38^++^ plasmablasts/plasma cells (Supplementary Figure 1, Tables 2, 3). Sequencing revealed that CD11c^hi^ IgD^+^ and IgD^−^ populations from SLE patients were polyclonal and utilized only two genes at a significantly different frequency than the naïve population (Figure 6A), e.g., V3–30 in CD11c^hi^ IgD^+^ B cells and V3–13 in both CD11c^hi^ IgD^+^ and IgD^−^ B cells. The V4–34 gene associated with autoimmunity (37) was highly expressed in CD11c^hi^ B cells, but not at a level different from the naïve population. However, when compared to memory B cells, both CD11c^hi^ IgD^+^ and IgD^−^ populations manifested greater variance in V-gene utilization with 3 genes over-expressed and 3 genes under-expressed (Figure 6B). When compared to circulating plasma cells, both CD11c^hi^ subsets showed 6 genes were over-expressed, including V4–34, and 2 V genes were under-represented (Figure 6C). This suggests that the CD11c^hi^ population contains a diverse repertoire similar to naïve, and they appear to lack significant selection against antigen.

### Somatic Hypermutation Frequencies Are Consistent With a GC Origin

**Figure 7:**
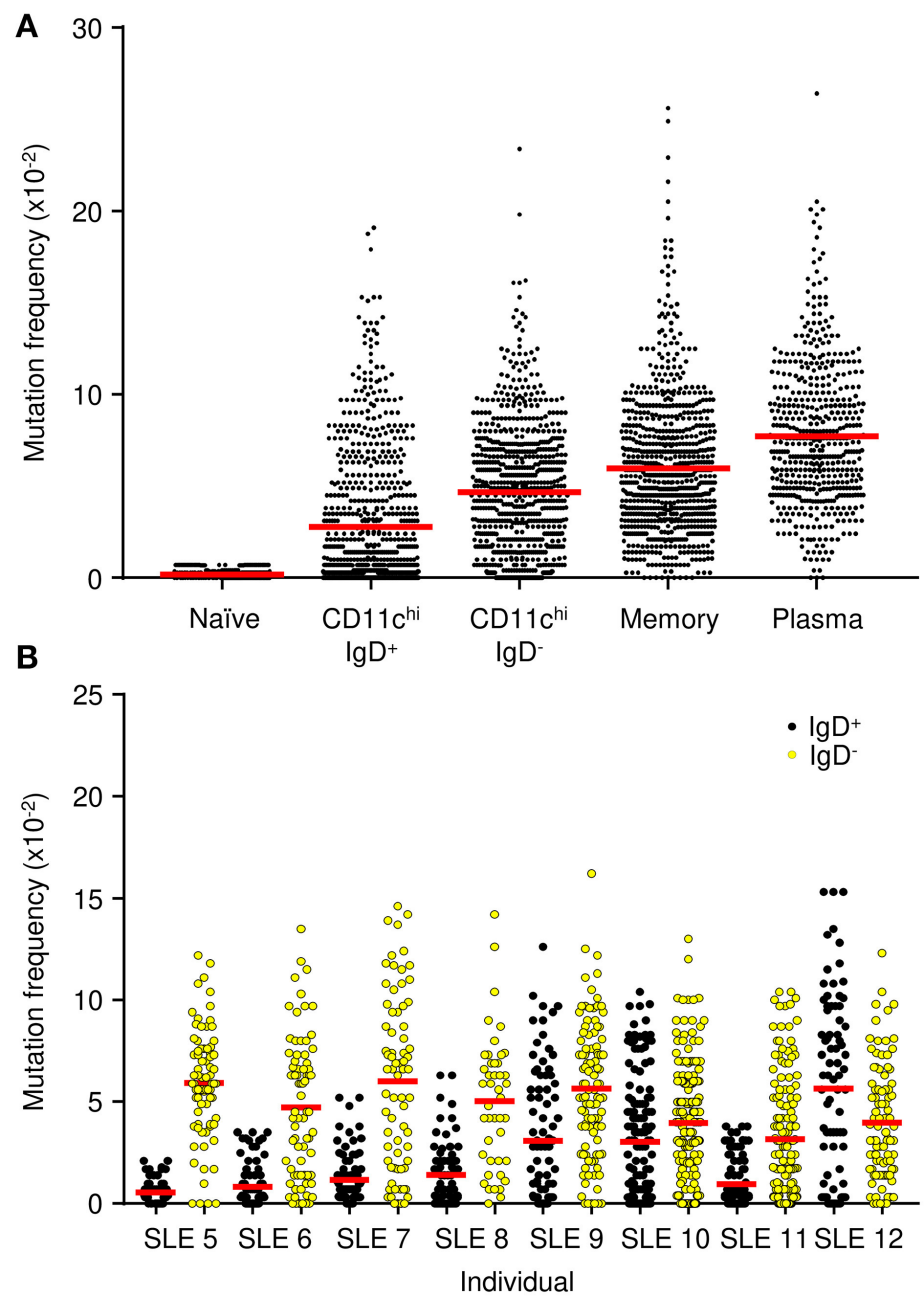
IgH V-gene mutation frequency. **(A)** Combined frequencies for each cell population from eight SLE patients. **(B)** Frequency for the CD11c^hi^ IgD^+^ (black) and CD11c^hi^ IgD^−^ (yellow) B cells from each individual SLE patient. Dots represent the mutation frequency for each IgH sequence, red line represents the average frequency of the population.

Mutation frequencies were measured to confirm active diversification of V_H_ genes. Naïve B cells had a frequency of 0.4 × 10^−2^ mutations/bp, which likely represents the PCR error rate (Figure 7A). The average mutation frequency in CD11c^hi^ B cells was substantially elevated for IgD^+^ sequences, 2.8 × 10^−2^, and IgD^−^ sequences, 4.7 × 10^−2^. The higher mutation frequency in V_H_ genes from IgD^−^ cells was confirmed in samples from most of the individual patients (Figure 7B), which supports the conclusion that switched cells undergo more mutation events (42). As a comparison, the mutational frequency in memory and plasma cells was even higher at 6.0 × 10^−2^ and 7.7 × 10^−2^, respectively (Figure 7A). Nonetheless, the data are consistent with the observation that both CD11c^hi^ IgD^+^ and IgD^−^ B cells have mutational frequencies typical of a GC experience.

### CD11c^hi^ Subsets Are Clonally Related to Each Other and to Memory and Plasma Cells

**Figure 8:**
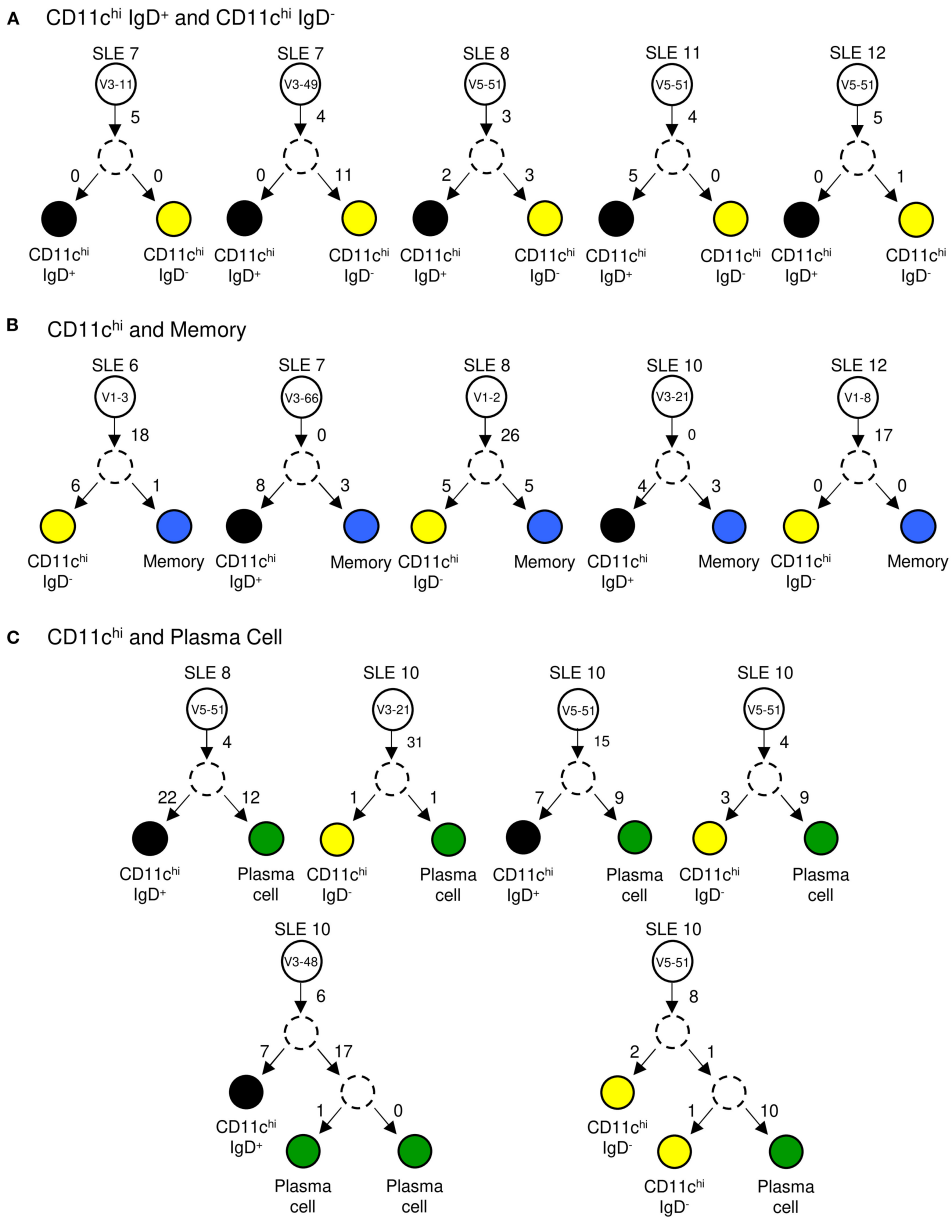
Clonal tree analysis. IgH V-genes with identical CDR3 nucleotide sequences were compared between two independently sorted populations from a single patient. Examples of shared sequences between: **(A)** CD11c^hi^ IgD^+^ (black circle) and CD11c^hi^ IgD^−^ (yellow circle) clones, **(B)** CD11c^hi^ IgD^+^ or IgD^−^ and memory (blue circle) B cell clones, and **(C)** CD11c^hi^ IgD^+^ or IgD^−^ and plasma cell (green circle) clones. White circles represent the naïve precursor labeled with its V-gene identity. Dashed circles represent a hypothetical intermediate cell which contained shared mutations before diverging. Numbers on arrows represent the number of mutations between cells.

To further confirm that CD11c^hi^ IgD^+^ and IgD^−^ B cell subsets from SLE are a common cell population with various stages of class switching, we analyzed the clonal relationship between the two populations. To create clonally-related trees, DNA sequences with identical V_H_ CDR3 nucleotide sequences were considered to be related and were grouped together. In each case, the common precursor had undergone somatic hypermutation with additional mutations occurring after the cells branched. The results in Figure 8A show that unique mutations occurred in both IgD^+^ and IgD^−^ branches. Since our analysis suggests that CD11c^hi^ B cells are GC-emigrants, we wanted to address whether they originate from the same precursor cell as memory and plasma cells. Comparing CD11c^hi^ to memory B cells, we identified several clonally-related cells, which were independent of IgD status (Figure 8B). Similar clonal relationships were seen with CD11c^hi^ and plasma cells (Figure 8C). Taken together, this analysis demonstrates that CD11c^hi^ B cells originate from the same precursor population as other antigen-experienced B cells.

## Discussion

Evidence that CD11c^hi^ B cells represent a distinct stage of B cell differentiation separate from naïve B cells or classical memory B cells comes from the thousands of DEGs compared to either of these populations, as well as the distinct separation of naïve, memory and CD11c^hi^ IgD^+^ and IgD^−^ B cells by PCA. A recent report using mass cytometry also shows clear separation of a similar CD19^hi^ CD11c^+^ population from other B cell subsets in healthy donors (43). Moreover, the B cell populations referred to as CD11c^hi^, anergic, atypical, and DN2 are more closely related to each other than to either naïve or memory B cells, suggesting that they represent B cells at similar, if not identical, stages of differentiation. Using a different procedure to identify these cells (CD27^−^ CXCR5^−^), Sanz and colleagues have suggested that there may be two different subpopulations based on IgD expression: IgD^+^ activated naïve (37, 38) and IgD^−^ DN2 cells (3).

Our current analysis employed a systems biology approach that did not rely on expression of a single gene to trace these populations, but instead looked at thousands of DEGs to delineate the transcriptional networks operating in each cell type. The results indicate that the populations are highly synonymous despite the differential expression of IgD. Notably, molecular analysis of the DEGs of CD11c^hi^ IgD^+^ and IgD^−^ B cells demonstrated large numbers of increased transcripts associated with the negative regulation of signaling through the B cell receptor, as well as increased transcripts of genes associated with lysosome, endosome and cytoskeletal proteins. This analysis showed that even though the CD11c^hi^ IgD^+^ B cells shared a few genes in common with naïve cells, in agreement with previous work (37), CD11c^hi^ IgD^+^ and IgD^−^ B cells were equally distant to both naïve and memory cells. Of note, there were 175 differentially expressed transcripts between CD11c^hi^ IgD^+^ and IgD^−^ B cells, which explains their individual grouping by PCA analysis. By hierarchical clustering, IgD+ and IgD- B cells were intermingled by patient rather than separating by IgD^−^ and IgD^+^ groups, and they were equally distant from naïve and memory B cells. This likely represents the differences in these graphical representations; PCA emphasizes differences to find the sources of variance between samples as opposed to hierarchical clustering that tries to form groups based on similarities. Therefore, the data suggest that these cells are virtually indistinguishable by gene expression profile, implying a comparable functional and differentiation status. Their Ig V_H_ repertoires were also analogous, and the V_H_ genes had similar levels of somatic hypermutation. Thus, these two populations are remarkably similar except for heavy chain isotype expression.

Transcriptomic analysis revealed a vast separation of expressed genes and cell signaling pathways between CD11c^hi^ cells and memory or plasma cells. It has been suggested that since CD11c^hi^ cells are antigen-experienced, they would share memory characteristics. Although they have increased transcripts for lysosome, endosome, and cytoskeletal genes, these may indicate increased antigen presentation capabilities. HLA class II transcripts were enriched as a group in CD11c^hi^ B cells, and may facilitate antigen presentation, which is consistent with data from the ABC population expressed in autoimmune-prone mice (44). Although B cells from humans and mice expressing CD11c have been considered to be ABCs, we directly examined how related they were by comparing DEGs and pathway analysis of increased and deceased transcripts compared to naïve B cells. Our results suggest that there is some overlap in cell markers, and both populations had increased B cell inhibitory signaling genes, but there were many differences in pathways between these two populations. The increase in multiple cytokines and chemokines by mouse ABC seemed to suggest a very different functional relevance. Functionally, ABCs respond to TLR stimulation while they are refractory to BCR and CD40 activation (24). However, CD11c^hi^ B cells proliferate rapidly in the presence of activated T cells or CD40 and BCR stimulation (1). Is there a CD11c^hi^ functional homolog in mice? Recent analysis has shown the presence of T-bet^+^ B memory cells, which are derived from the GC (45). A small subset of these cells expressed CD11c and may be a functional equivalent to human CD11c^hi^ B cells. Further studies are needed to determine whether the two cell types have analogous functions.

Notably, CD11c^hi^ B cells shared only a few transcripts with GC cells, suggesting they are no longer participating in these processes. The over-expression of some GC genes, such as *AICDA*, in comparison to memory B cells may indicate a more recent emigration from the GC. Analysis of mutation frequencies clearly showed that V_H_ genes of CD11c^hi^ B cells were highly mutated compared to naïve B cells, consistent with a GC experience, although they were not as mutated as either memory B cells or plasma cells, implying a truncated GC transit. Consistent with an origin within the GC, CD11c^hi^ B cells have a shared clonal ancestor as memory and plasma cells which are byproducts of the GC. Reports also suggest that CD11c^hi^ cells are poised to become plasma cells (1, 3). However, we found minimal shared transcriptional networks with plasma cells, such as unfolded protein response, increased endoplasmic reticulum, Golgi, and Ig transcripts. Thus, CD11c^hi^ B cells appear to be derived from the GC under inflammatory conditions, and they are transcriptionally positioned between GC B cells and plasma cells.

IgH V gene analysis revealed that even though CD11c^hi^ B cells were not different in their repertoire compared to naïve B cells, they were significantly different from both memory and plasma cells. This suggests there is something different about the selection process for CD11c^hi^ B cells. For example, the IgH V4–34 gene associated with autoimmune antibodies has been reported to be excluded or negatively selected in GC (46), but our repertoire analysis confirmed that the V4–34 gene was enriched in CD11c^hi^ B cells compared to the memory and plasma cell populations. One possible explanation is that CD11c^hi^ B cells expressing autoimmune V genes are not properly negatively selected in the GC, accounting for their enrichment in autoantibody production in memory and plasma cells (1, 3).

Indeed, the most startling characteristic of the CD11c^hi^ subset is its resistance to stimulation through the BCR. The population has increased transcripts associated with inhibitory PTP, inhibitory FCRL, inhibitory DUSP and inhibitory signaling adaptors, indicating that they are hyporesponsive to BCR stimulation. Rather than being deleted, CD11c^hi^ B cells may have survived and exited the GC as autoreactive B cells that are refractory to BCR signaling. Dampened BCR signaling is a shared characteristic of atypical memory cells from malaria patients (21), CD21^low^ B cells from RA or CVID patients (7), and CD21^−^ SYK^high^ B cells from HIV, CVID and SLE patients (47). These cells now represent a threat, in that if they receive a rescue signal by bystander help through another receptor such as CD40 or TLR7, they can differentiate into autoantibody-producing plasma cells (1, 3, 4). Future efforts to control their activation may mitigate progression of disease.

## Data Availability Statement

The datasets generated for this study can be found in online repositories. The names of the repository/repositories and accession number(s) can be found in the article/Supplementary Material.

## Ethics Statement

The studies involving human participants were reviewed and approved by the Institutional Review Board of the National Institute of Arthritis and Musculoskeletal and Skin Diseases (protocol 94-AR-0066, and 00-AR-0222, respectively). The patients/participants provided their written informed consent to participate in this study.

## Author Contributions

The concept was conceived by RE, PL, and PG. The overall study design was developed by VK, RM, and MC. Additional experiments were performed by WY, SW, PB, and AG. Human samples were provided by SH. Manuscript was written by RM, MC, RE, PL, and PG. All authors contributed to the article and approved the submitted version.

## Conflict of Interest

MDC, PB, ACG, and PEL are employed by AMPEL BioSolutions, LLC. VK is a full-time employee and shareholder of AstraZeneca. RE and SWare full time employees and shareholders of Viela Bio, and shareholders of AstraZeneca. The remaining authors declare that the research was conducted in the absence of any commercial or financial relationships that could be construed as a potential conflict of interest.
