# Supplementary material for: Transcriptome and IgH Repertoire Analyses Show That CD11chi B Cells Are a Distinct Population With Similarity to B Cells Arising in Autoimmunity and Infection
Source: Front Immunol. 2021 Mar 19;12:649458. doi: 10.3389/fimmu.2021.649458 (PMC8017342; doi:10.3389/fimmu.2021.649458)
Supplement: Supplementary file 2 [file Data_Sheet_2.pdf]

Table S1. Demographics and clinical characteristics of SLE patients examined for RNA transcriptional analysis

Demographics			
Total patient number	4	C3, mg/dL (median [range])	108.4 [45.3-118.5]
Gender (% Female)	100	C4, mg/dL (median [range])	15.3 [5.6-18.6]
Age (median [range])	47.5 [26-57]	ESR, mm/hr (median [range])	13 [5-91]
SLEDAI (median [range])	2 [0-11]	CRP, mg/L (median [range])	2.7 [1.1-3.7]

Clinical Characteristics

Auto-antibodies		Medication	
anti-DNA (% positive)	100	prednisone (number low < 10 mg)	3
anti-nuclear, ANA (% positive)	75	prednisone (number high > 50 mg)	1
Lupus anti-coagulant (% positive)	0	hydroxychloroquine (number)	4
extractable nuclear antigen (% positive)	75	azathioprine	1
		mycophenolate mofetil (number)	1
		methotrexate (number)	1
		biologics (number, belimumab)	1

Table S2. Demographics and clinical characteristics of SLE patients examined for IgH repertoire analysis

Demographics

Total patient number	8	C3, mg/dL (median [range])	103.4 [79.6-119.4]
Gender (% Female)	100	C4, mg/dL (median [range])	13.7 [5.4-21.4]
Age (median [range])	51.5 [28-73]	ESR, mm/hr (median [range])	21 [13.0-58.0]
SLEDAI (median [range])	3 [0-8]	CRP, mg/L (median [range])	2.3 [1.0-64.8]

Clinical Characteristics

Auto-antibodies		Medication	
anti-DNA (% positive)	62.5	prednisone (number low < 10 mg)	5
anti-nuclear, ANA (% positive)	100	prednisone (number high > 50 mg)	2
Lupus anti-coagulant (% positive)	0	hydroxychloroquine (number)	5
extractable nuclear antigen (% positive)	62.5	azathioprine	3
		mycophenolate mofetil (number)	1
		methotrexate (number)	2
		biologics (number, belimumab)	1

Table S3. IgH sequencing information

Clones sequenced

Patient ID	naïve	CD11c^{Hi} IgD^{+}	CD11c^{Hi} IgD^{-}	memory	plasma cell
SLE 5	136	75	80	95	---
SLE 6	---	94	74	84	---
SLE 7	---	95	72	81	85
SLE 8	---	83	44	69	76
SLE 9	---	87	104	89	---
SLE 10	184	164	174	161	151
SLE 11	115	115	147	83	143
SLE 12	57	76	94	126	68
Total	492	789	789	788	523

Nucleotides sequenced

Patient ID	naïve	CD11c^{Hi} IgD^{+}	CD11c^{Hi} IgD^{-}	memory	plasma cell
SLE 5	38979	21840	22952	27334	---
SLE 6	---	27048	22128	24102	---
SLE 7	---	27360	20745	23349	24493
SLE 8	---	23915	12672	19842	21898
SLE 9	---	25318	29919	25900	---
SLE 10	53001	48138	52480	46385	43523
SLE 11	34797	32759	42334	24216	41985
SLE 12	16391	23082	27013	36243	19581
Total	143168	229460	230243	227371	151480

Mutations

Patient ID	naïve	CD11c^{Hi} IgD^{+}	CD11c^{Hi} IgD^{-}	memory	plasma cell
SLE 5	199	328	1403	1817	---
SLE 6	---	433	1039	1094	---
SLE 7	---	739	1247	1807	2172
SLE 8	---	707	637	1309	1977
SLE 9	---	861	1693	1316	---
SLE 10	221	1679	2099	2829	3066
SLE 11	117	485	1439	1453	2993
SLE 12	47	1305	1189	1946	1446
Total	584	6537	10746	13571	11654
