# Supplementary figures and images for: Transcriptome and IgH Repertoire Analyses Show That CD11chi B Cells Are a Distinct Population With Similarity to B Cells Arising in Autoimmunity and Infection
Source: Front Immunol. 2021 Mar 19;12:649458. doi: 10.3389/fimmu.2021.649458 (PMC8017342; doi:10.3389/fimmu.2021.649458)

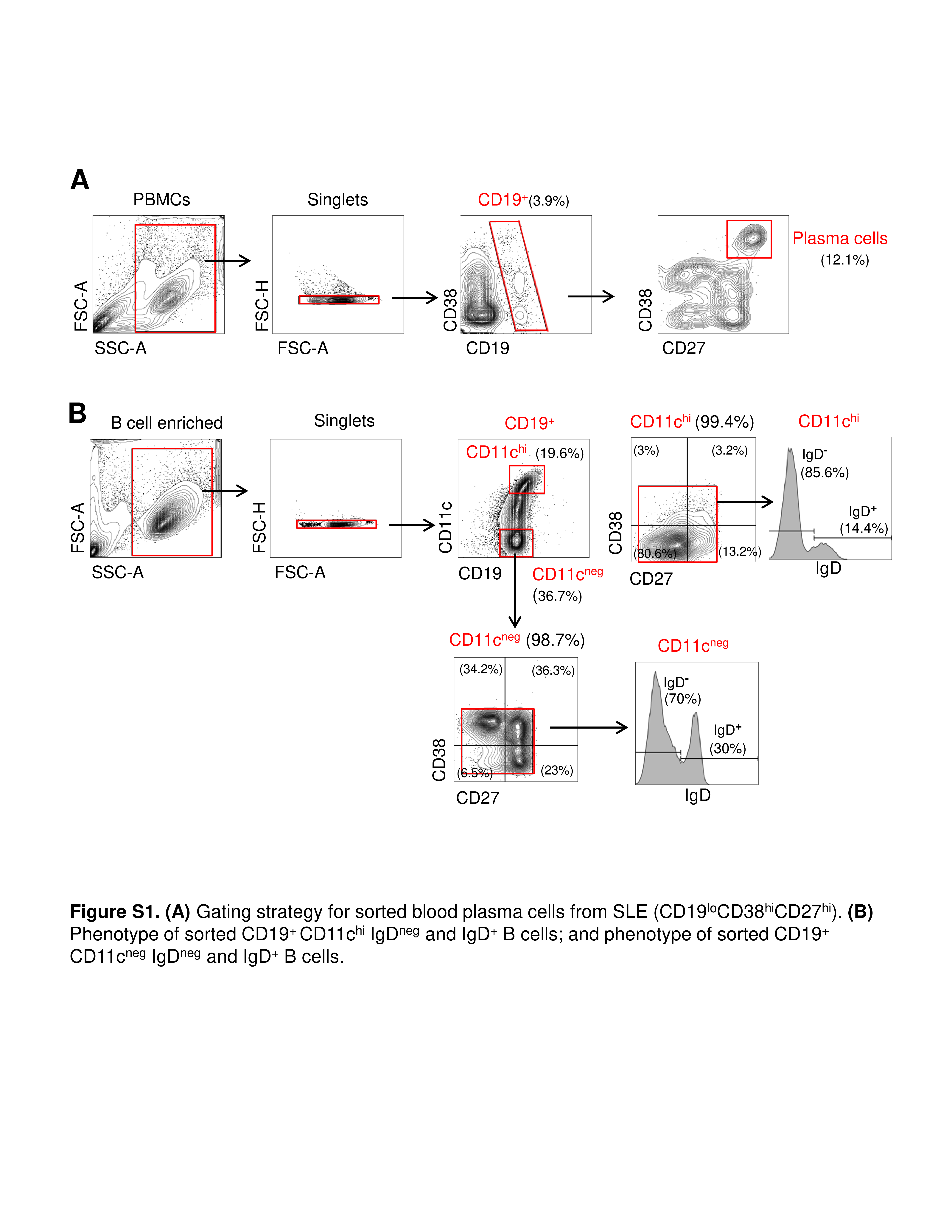

Supplement: Supplementary file 3 [file Image_1.TIF]

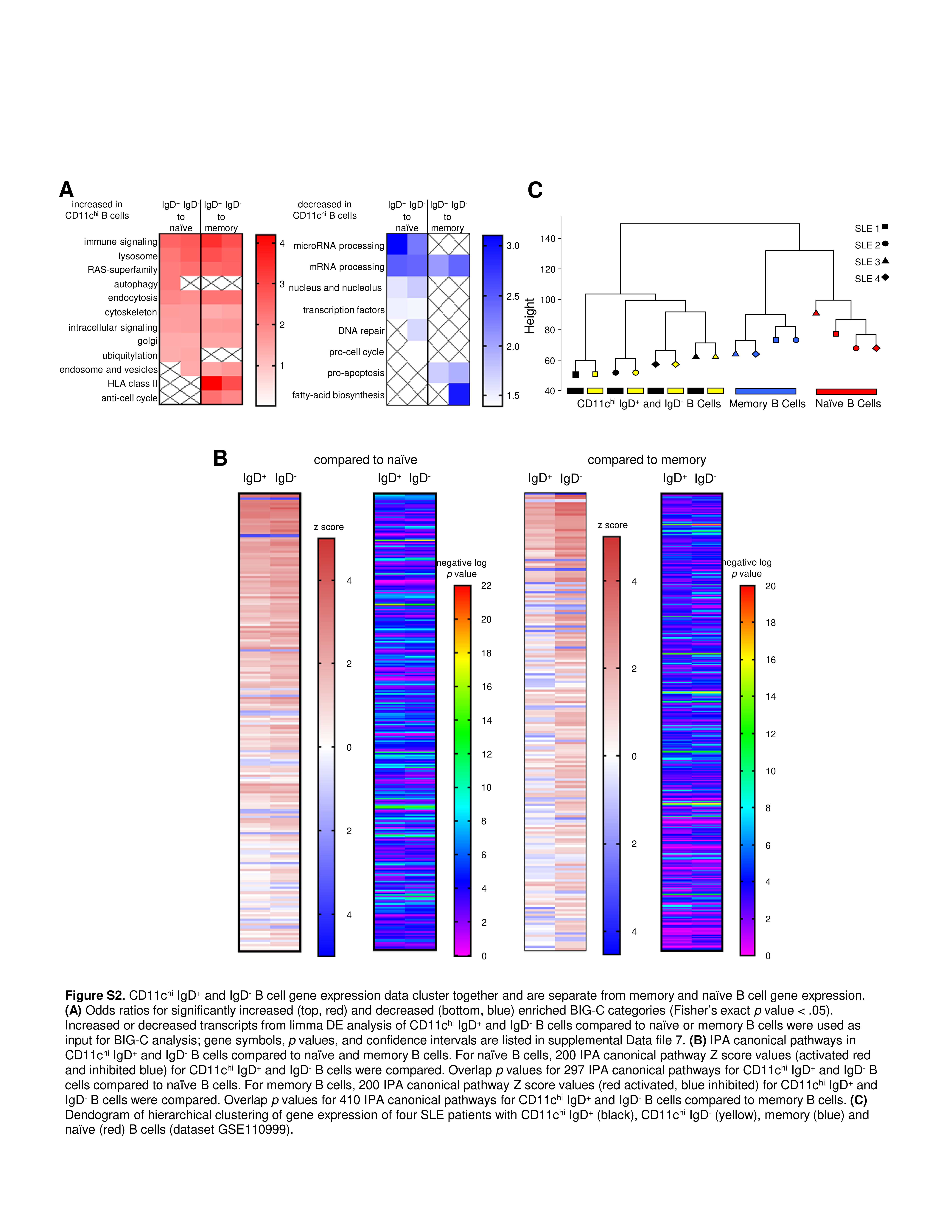

Supplement: Supplementary file 4 [file Image_2.TIF]
